# Personalization variables in digital mental health interventions for depression and anxiety in adolescents and youth: a scoping review

**DOI:** 10.3389/fdgth.2025.1500220

**Published:** 2025-05-15

**Authors:** Vajisha Udayangi Wanniarachchi, Chris Greenhalgh, Adrien Choi, James R. Warren

**Affiliations:** ^1^ School of Computer Science, University of Auckland, Auckland, New Zealand; ^2^ School of Computer Science, University of Nottingham, Nottingham, United Kingdom

**Keywords:** personalisation, digital mental health interventions, adolescents, youth, anxiety, depression, adherence

## Abstract

**Introduction:**

The impact of personalization on user engagement and adherence in digital mental health interventions (DMHIs) has been widely explored. However, there is a lack of clarity regarding the prevalence of its application, as well as the dimensions and mechanisms of personalization within DMHIs for adolescents and youth.

**Methods:**

To understand how personalization has been applied in DMHIs for adolescents and young people, a scoping review was conducted. Empirical studies on DMHIs for adolescents and youth with depression and anxiety, published between 2013 and July 2024, were extracted from PubMed and Scopus. A total of 67 studies were included in the review. Additionally, we expanded an existing personalization framework, which originally classified personalization into four dimensions (content, order, guidance, and communication) and four mechanisms (user choice, provider choice, rule-based, and machine learning), by incorporating non-therapeutic elements.

**Results:**

The adapted framework includes therapeutic and non-therapeutic content, order, guidance, therapeutic and non-therapeutic communication, interfaces (customization of non-therapeutic visual or interactive components), and interactivity (personalization of user preferences), while retaining the original mechanisms. Half of the interventions studied used only one personalization dimension (51%), and more than two-thirds used only one personalization mechanism. This review found that personalization of therapeutic content (51% of the interventions) and interfaces (25%) were favored. User choice was the most prevalent personalization mechanism, present in 60% of interventions. Additionally, machine learning mechanisms were employed in a substantial number of cases (30%), but there were no instances of generative artificial intelligence (AI) among the included studies.

**Discussion:**

The findings of the review suggest that although personalization elements of the interventions are reported in the articles, their impact on younger people's experience with DMHIs and adherence to mental health protocols is not thoroughly addressed. Future interventions may benefit from incorporating generative AI, while adhering to standard clinical research practices, to further personalize user experiences.

## 1 Introduction

Mental disorders are characterized by disruption in an individual's thinking patterns, emotional control, or actions associated with distress or impaired functioning (1). They constitute a significant portion of the worldwide health burden, as noted by Wies et al. (2), a claim consistent with global health data indicating that 1 in every 8 people (nearly 1 billion people) were living with a mental disorder in 2019 (1) and meta-analysis showing mental disorders are associated with a median life expectancy loss of 10 years (3) While estimates vary, previous syntheses have consistently reported high global prevalence rates of mental health disorders among adolescents (10–19 year olds) (4, 5). Evidently, approximately 1 in 7 adolescents experience a mental disorder, contributing to around 15% of the global burden of disease in this age group (6). Comparably, mental health and wellbeing of university students has become an important public health concern (7) with suicide ranking as the third leading cause of death among youth (15–24-year-olds). Specifically, during the COVID-19 pandemic, prevalence of depression among college students was 39%, and depression and anxiety were the most commonly reported psychological issues among them in studies on the mental health impact of the new coronavirus (8). Consequently, depression and anxiety have become the primary contributors to disability in youth (9). However, such mental health issues remain underdiagnosed and undertreated among adolescents and youth (10).

Mental health challenges can profoundly influence the developmental journey of adolescents (10), exerting a lasting effect on their health and social integration as adults (11). Hence, addressing mental health challenges in adolescents and youth early on could mitigate the repercussions of carrying these issues into adulthood. Nonetheless, a staggering 75% of adolescents grappling with mental health issues do not engage with mental health services (12), primarily due to their reluctance to seek assistance (13, 14). This hesitancy stems from attitudinal barriers, including a lack of perceived necessity for treatment, fears of judgment, limited understanding of mental health, and a strong inclination toward self-management (15–17).

The growing utilization of the internet and digital platforms in recent years presents substantial opportunities to meet the mental health needs of adolescents and youth (18). Digital technologies, particularly those that are readily accessible, offer practical solutions to expand the reach of evidence-based interventions for both adolescents and adults (19–21).

Converting psychosocial interventions into digital formats, known as digital mental health interventions (DMHIs), has the potential to surmount existing obstacles to traditional care, thereby enhancing access to mental health support and resources (22). While certain DMHIs have demonstrated effectiveness comparable to traditional mental health services like psychotherapy and pharmacotherapy in addressing mental health conditions (23, 24), sustaining individuals' engagement can be challenging as they contend with various demands in their daily lives, leading to issues of poor adherence (25, 26). To address these engagement and adherence challenges, researchers have explored strategies that make DMHIs more relevant and engaging for users.

One such strategy is personalization, which involves tailoring digital interventions to individual users to enhance relevance and engagement. Personalization, as defined by Blom and Monk (27), is “the process that changes the functionality, interface, information content, or distinctiveness of a system to increase its personal relevance to the individual”. It has been used widely in emerging digital technologies in fields such as marketing (28), education (29) and health (30). For adolescents and young people, personalization is particularly important due to their distinct cognitive, emotional, and social developmental stages, which influence how they interact with digital interventions (31). Their engagement with DMHIs is shaped by factors such as digital literacy, peer dynamics, and the need for developmentally appropriate and relatable content (32). Additionally, as they navigate identity formation and evolving mental health challenges, interventions that align with their preferences, values, and socio-cultural contexts are more likely to be effective. Consequently, personalizing population-wide health interventions by considering both general health guidelines and individual user characteristics can enhance their relevance, engagement, and effectiveness (33, 34). A common objective of personalized digital interventions has been to achieve behavioral change (34). Personalization has been demonstrated to reduce users' cognitive load (35), increase persuasion and enhance user satisfaction (36), thereby improving the effectiveness of personalized health behavior interventions (37). Therefore, personalization can be considered as a strategy that enhance user experience and engagement in DMHIs (38, 39).

Despite the numerous personalization variables proposed or implemented in DMHIs in recent years, including user characteristics, content characteristics and therapy activities (40–42), there has been a lack of in-depth exploration into the application of these personalization variables in DMHIs designed for younger people. Hence, this scoping review seeks to examine the evidence of personalization variables reported in DMHIs tailored for adolescents and youth, addressing the following research question. As depression and anxiety rank among the primary causes of disability in adolescents, this review will focus specifically on DMHIs tailored for adolescents and youth grappling with anxiety and depression.

RQ: Which personalization variables are utilized in DMHIs designed for adolescents and youth?

## 2 Method

The objective of this scoping review is to investigate the research question outlined in the introduction by gathering information on personalization variables employed in DMHIs for adolescents and youth. To achieve this goal, a search was conducted on July 01, 2024, across two databases, namely PubMed and Scopus, to identify relevant publications.

### 2.1 Search strategy

**Figure 1:**
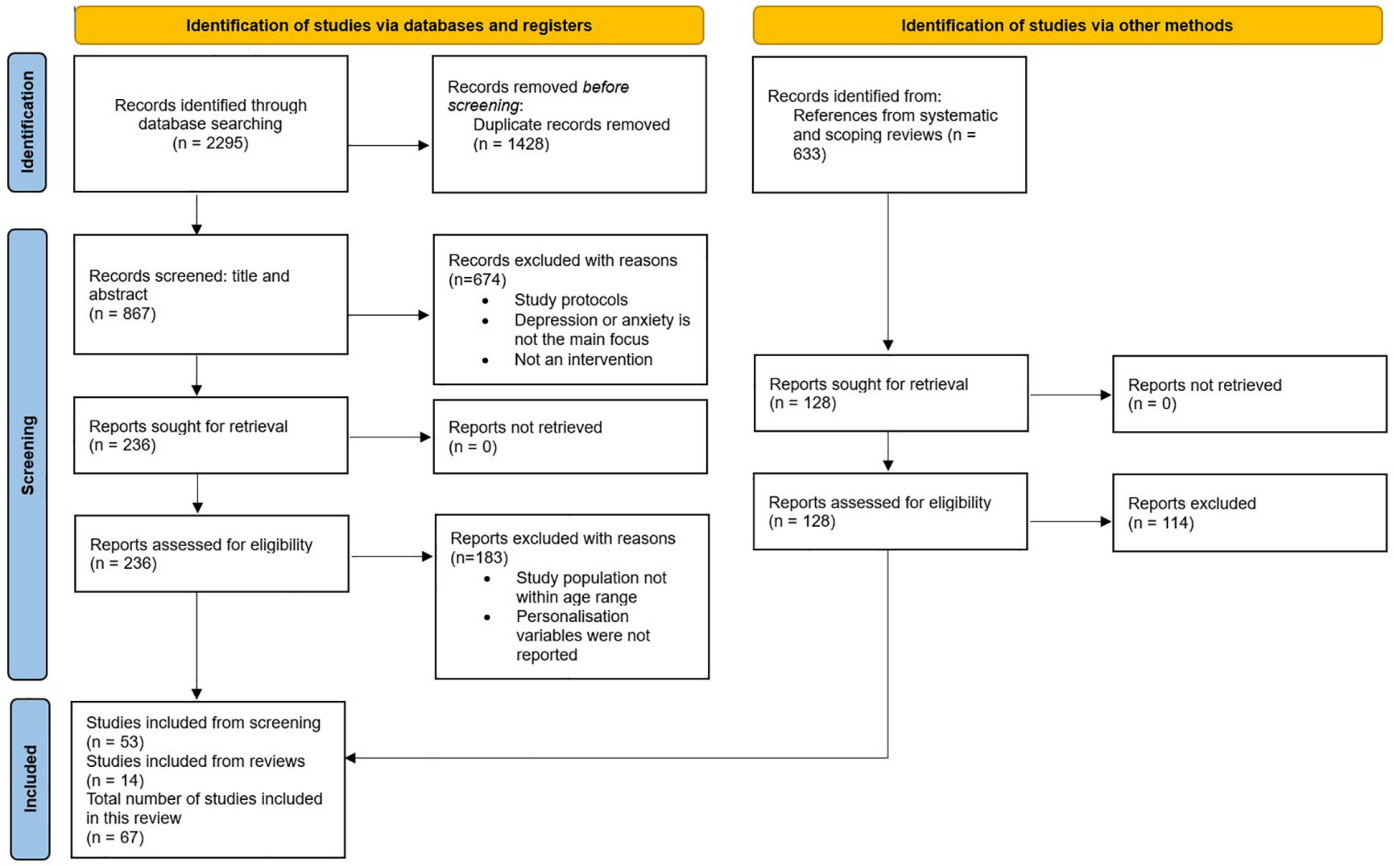
PRISMA flow chart.

To ensure a thorough exploration of the research domain, a systematic search strategy was devised. This strategy encompassed three key concepts: depression and anxiety, digital mental health interventions, and adolescents and young individuals. Using search queries outlined in Table 1, relevant publications were retrieved. The searches across databases yielded 2,295 results, from which 867 unique sources were identified for further screening following the removal of duplicates (refer to Figure 1).

**Table 1:** Search queries.

Database	Search query
PubMed	((“adolescent”[MeSH Terms]) OR (adolesce*[Title/Abstract]) OR (adolescent[Title/Abstract]) OR (“young”[Title/Abstract]) OR (“youth”[Title/Abstract])) AND ((“mental health”[MeSH Terms]) OR (“depression”[MeSH Terms]) OR (“anxiety”[MeSH Terms]) OR (“mental health”[Title/Abstract]) OR (depress*[Title/Abstract]) OR (“anxiety”[Title/Abstract])) AND ((“digital intervention”[Title/Abstract]) OR (“e therapy”[Title/Abstract]) OR (“apps”[Title/Abstract]) OR (app[Title/Abstract]))
Scopus	(TITLE-ABS-KEY(ADOLESCENT) OR TITLE-ABS-KEY(adolesce*) OR TITLE-ABS-KEY(adolescent) OR TITLE-ABS-KEY(“young”) OR TITLE-ABS-KEY(“youth”)) AND (TITLE-ABS-KEY(“mental health”) OR TITLE-ABS-KEY(depression) OR TITLE-ABS-KEY(anxiety) OR TITLE-ABS-KEY(“mental health”) OR TITLE-ABS-KEY(depress*) OR TITLE-ABS-KEY(anxiety)) AND (TITLE-ABS-KEY(“digital intervention”) OR TITLE-ABS-KEY(“e therapy”) OR TITLE-ABS-KEY(apps) OR TITLE-ABS-KEY(app))

### 2.2 Study selection

Following the PRISMA statement and flowchart (43), our review process comprised four phases: identification, screening, eligibility assessment, and final synthesis. Initially, articles meeting the following criteria were considered for retrieval: (a) peer-reviewed journal publications, (b) written in English, and (c) published between January 2013 and July 2024. From this initial retrieval, 1,428 duplicates were identified and removed. Subsequently, the remaining articles underwent screening based on their title and abstract, resulting in the extraction of 236 articles. Non-empirical papers, such as study protocols, were excluded during this stage, along with articles not primarily focused on depression and/or anxiety interventions or studies that were not carried out as an intervention. In this study we framed DMHIs as interventions that deliver mental health support primarily through digital platforms such as mobile applications, websites or chatbots. To ensure that only digital interventions were included, we excluded studies focusing solely on traditional face-to-face therapy, paper-based self-help materials, or interventions without a digital component. Further eligibility criteria included the age range of the study population [10–24 years; adolescents (10–19-year-olds) and young people (15–24-year-olds)] and the presence of personalization features within the intervention, particularly those that could impact usability. Ultimately, 53 articles met all inclusion criteria and were included in the final synthesis.

Apart from adhering to the systematic study selection criteria, the review articles obtained during the initial retrieval were examined to identify any additional relevant studies. Through this, 14 additional articles meeting the inclusion criteria were identified and subsequently included in the final synthesis.

### 2.3 Charting the data

A total of 67 articles were incorporated into the final synthesis, and a comprehensive review of the full-text articles was conducted. The initial extraction of study data was conducted by a single researcher (VW), after which two reviewers (VW and AC) independently screened the data. Final decisions were made through the agreement of both VW and AC. The extracted information included sample characteristics, research design, concepts/technology of intervention utilized, application name, personalization variables employed and the country where the intervention was conducted.

### 2.4 Inter-rater agreement

During the title and abstract screening phase, two reviewers screened 867 records, reaching agreement on 851 records (98.2% agreement rate). Discrepancies were resolved through discussion.

In the data extraction phase, two reviewers independently extracted the personalization variables reported in the 67 included studies, after which the results were compared. For 49 studies (73.1%), there was full agreement on the extracted personalization variables. In 14 studies (20.9%), initial differences in interpretation were resolved through discussion. In 4 studies (6.0%), partial agreement was reached when multiple personalization elements were present and interpretations varied. All cases were reviewed and consensus reached through discussion.

### 2.5 Conceptual framework

This study attempts to classify the personalization variables investigated in DMHIs in the recent past. We adopt the conceptual framework proposed by Hornstein et al. (44) which provides a range of categories both in terms of what is personalized (content, order, guidance and communication) and the mechanisms of personalization (user choice, provider choice, rule-based and by machine-learning model) in DMHIs. However, their framework excluded our customization and interactivity dimensions of personalization as they limited their framework to mechanisms affecting the *therapeutic* content and structure. Yet, non-therapeutic elements, such as changing the appearance of an avatar or goal setting, could enhance user engagement and adherence. For example, Hollis et al. (45) argue that the capacity for a young person to personalize minor aspects such as the gender and appearance of a “guide” might influence their perception and connection with a digital health intervention. Additionally, Birk and Mandryk (46) highlighted that avatar customization could increase enjoyment, effort invested, and time spent in a game-based intervention. Furthermore, it is evident that the use of interactivity can enhance engagement with digital interventions (47). Therefore, we have extended the conceptual framework proposed by Hornstein et al. (44) with the consideration of non-therapeutic elements such as user-driven tailoring and interactivity. This better aligns the framework with Blom and Monk (27) to more fully address the functionality, interface and distinctiveness aspects of their definition of personalization.

The derived framework includes six potential dimensions of personalization. Both therapeutic content delivered during a session and non-therapeutic content, such as information on mental health resources, will be considered to define the “content” dimension. The order of the sessions included in the intervention and the guidance provided by the providers will also be considered as therapeutic dimensions. Additionally, therapeutic communication, such as prompts and mechanisms targeting the timing of the intervention, and non-therapeutic communication, such as maintaining a personalized contact list, will be considered to define the “communication” dimension. To represent aspects of application personalization, absent from the earlier framework, “interface” and “interactivity” will be included as two additional dimensions. The interface dimension is defined as the customization of non-therapeutic content of the intervention, such as the visual appearance of avatars and application background. The interactivity dimension includes the personalization of user preferences in terms of application control and input, such as what data to share with the application, personalized methods of input, saving favorites, and replaying users' personal goals.

In terms of mechanism of personalization, this study uses the same four categories as (44). While this study primarily focuses on digital interventions, the most common personalization mechanism—User Choice—will be considered. Provider Choice will represent the mechanism of providing personalized feedback or suggestions by system moderators, therapists, or professional experts. For automated personalization mechanisms, rule-based and machine learning approaches will be considered as distinct mechanisms applying static (designer/expert-specified) or data-derived criteria for personalization, respectively.

**Figure 2:**
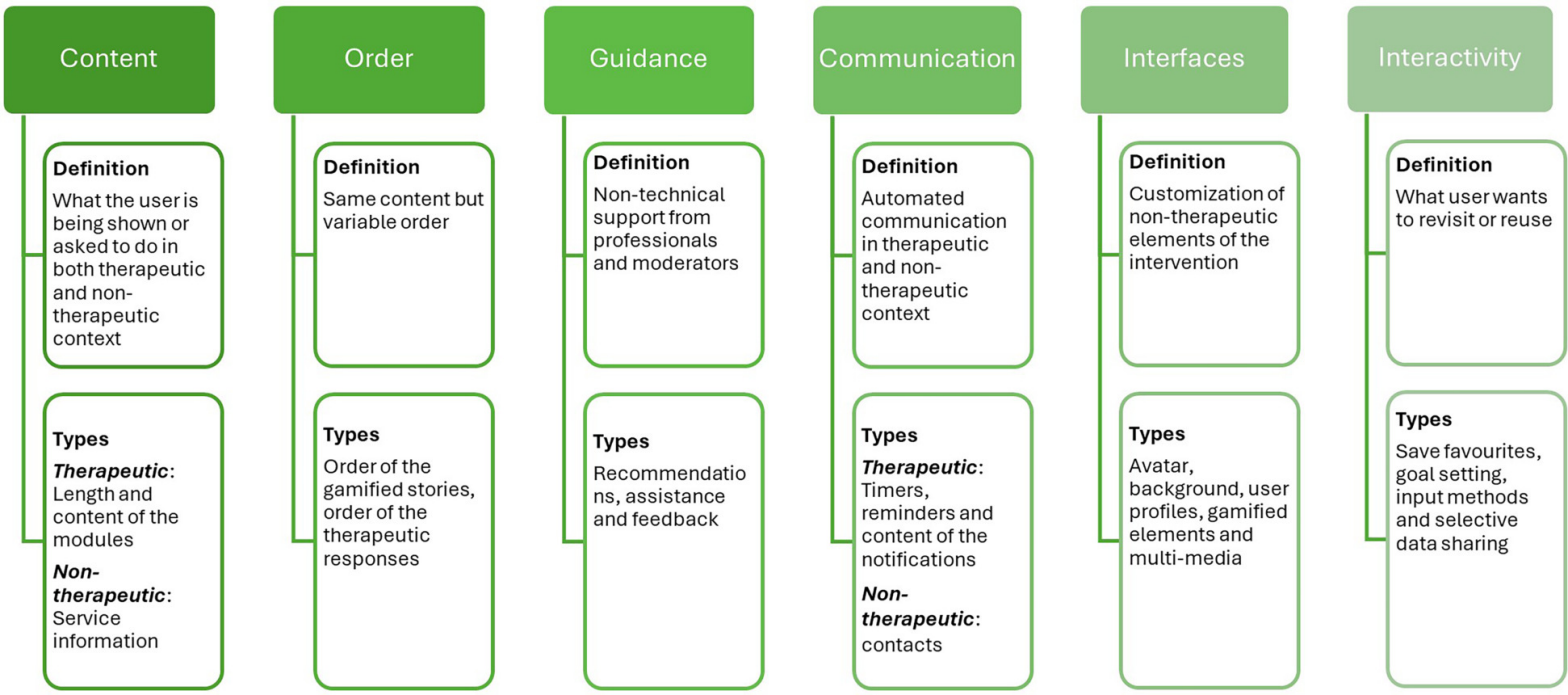
Personalization dimensions (what is personalized).

**Figure 3:**
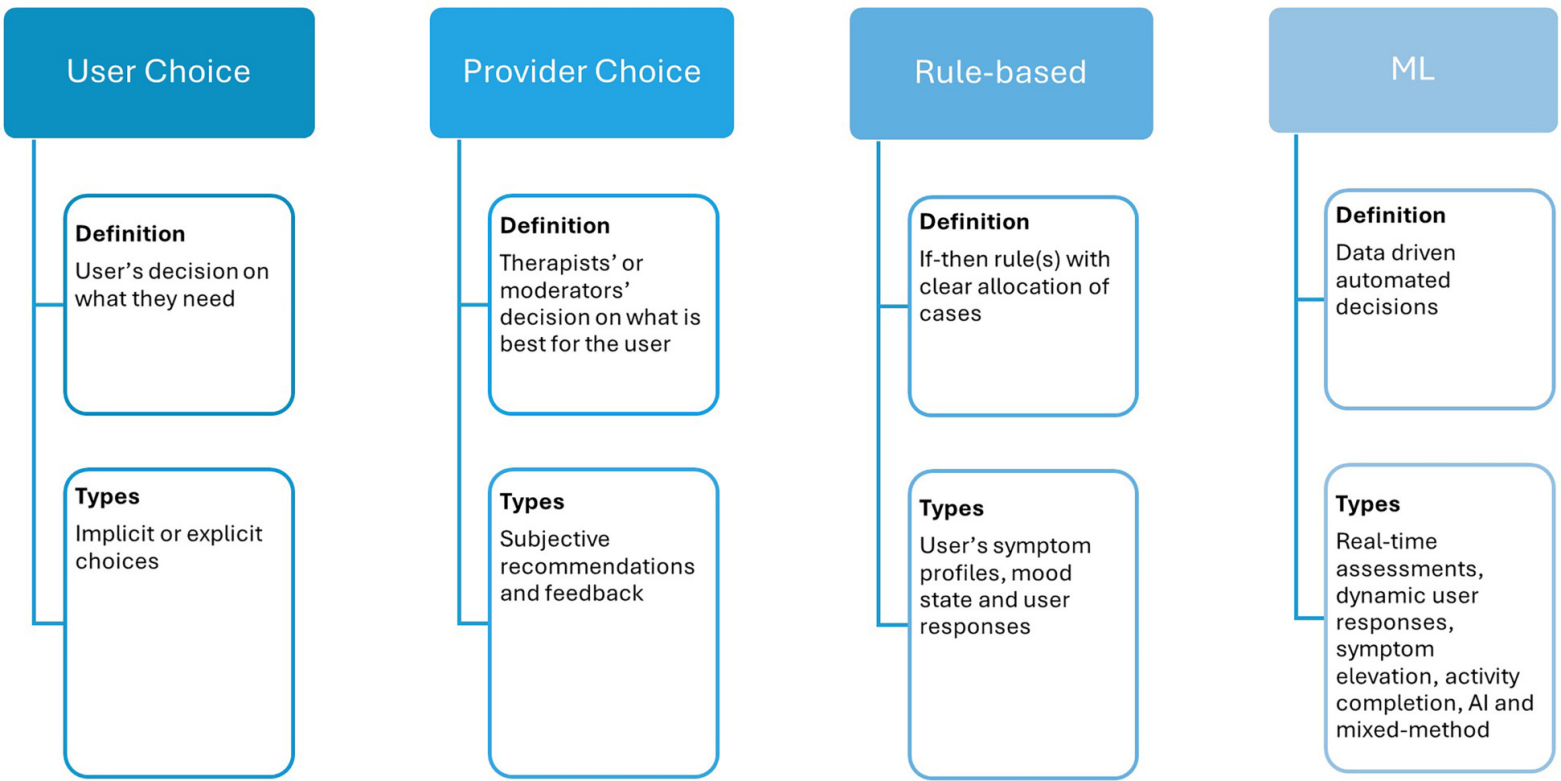
Personalization mechanisms (how it is personalized).

The definitions and types of personalization dimensions and mechanisms are summarized in Figure 2 and Figure 3, respectively.

## 3 Results

The selected 67 articles were analyzed to extract study characteristics and personalization variables. The extracted information of the reviewed articles is summarized in Table 2.

**Table 2:** Extracted information of the reviewed articles.

Authors	Research Design	Specific Sample Characteristics	Mobile/Web Application	Concepts Applied in the App	Country	Personalization Dimension	Personalization Mechanism
Almeqbaali et al. (75)	Mixed methods	University students	Haddy	Games, mood tracking	UAE	**Content (therapeutic)**: Duration of breathing exercises **Guidance**: Exercise suggestions	**User choice** **Machine learning**: Based on heartrate
Atik et al. (76)	Uncontrolled trial	University students	Elona therapy	CBT	Germany	**Content (therapeutic):** Activities, exercises and psychoeducational resources **Interactivity:** Personal toolkit of preferred content and tasks within the app	**Rule-based:** Based on respective symptoms, patient's needs, diagnosis, therapy progress and joint therapy decisions **Provider choice:** Track individuals progress and display personalized tasks and contents in the user's app **User choice**
Au-Yeung et al. (72)	Qualitative	Indigenous youth	JoyPop	Resilience theory	USA	**Communication (therapeutic)**: Timer to play sleep sounds **Communication (non-therapeutic)**: Social contacts	**User choice**
Bell et al. (103)	RCT	General adolescent and youth population	Mello	Just-in-time adaptive interventions model	Australia	**Content (therapeutic)**: Personalized therapy activities	**Machine learning**: Real-time assessment
Bidargaddi et al. (60)	RCT	General adolescent and youth population	ReachOut.com (Tool-Box)	Companion app	Australia	**Guidance**: Mental health app recommendations	**Machine learning**: Based on the user's selection of goals and answers to interactive quizzes
Birrell et al. (93)	RCT	Secondary school students	Mind Your Mate	Peer support, help seeking, psychoeducation, gamification	Australia	**Content (therapeutic)**: Mood tracking and self-care activities **Interfaces**: Customized avatar selection/color scheme/gamification content	**User choice**
Brooks et al. (112)	Mixed methods	General adolescent and youth population	Digital application of IMPEtUS	Mental health literacy, Games	Indonesia	**Interfaces:** Customization of the character of the game	**User choice**
Bruhns et al. (77)^†^	RCT	University students	MCT & More	CBT, mindfulness, acceptance and commitment therapy, metacognitive training	Germany	**Content (therapeutic)**: User's own exercises	**User choice**
Burn et al. (90)	User-centred design	Secondary school students	Artemis-A	Computerized adaptive testing	UK	**Content (therapeutic)**: Personalized assessment, tailored reports	**Machine learning**: Selecting assessment item based on user's response to the preceding item, tailored reports based on automated scoring
Chen et al. (104)	Pilot study	Not defined	ExpDepression	PHQ-9	USA	**Content (therapeutic):** Modified text based PHQ-9 **Guidance:** Automatically categorize user into risk categories and reported to a care team or helpline	**Provider choice:** Modified PHQ-9 based on provider's assessment of patient **Rule based:** Risk categories based on patient's mood, PHQ-9 score and self-harm ideation
Christie et al. (73)	Co-design	General adolescent and youth population	Quest—Te Whitianga	Gamification, CBT, mindfulness	New Zealand	**Interfaces**: Customized gamified elements such as “home island”, customized avatar	**User choice**
Cliffe et al. (52)	Mixed methods	University students	BlueIce	CBT, dialectical behavioral therapy	UK	**Content (therapeutic):** Personalized mood-lifting activities **Communication (non-therapeutic):** Contact list	**User choice**
Dietvorst et al. (48)	Experimental study	General adolescent and youth population	The Grow It!	CBT, games	Netherlands	**Content (therapeutic)**: Choose one of three unique challenges each day **Interfaces**: Customized user profiles and nicknames	**User choice**
Dietvorst et al. (49)	Pre-post study	General adolescent and youth population	The Grow It!	CBT, Experience Sampling Method (ESM)	Netherlands	**Interfaces**: Mood profiles	**Machine learning**: Based on self-reported mood in the ESM
Dietvorst et al. (50)	Longitudinal Study	General adolescent and youth population	The Grow It!	CBT, Games	Netherlands	**Content (therapeutic):** Choose one of three unique challenges each day **Interfaces:** Customized user profiles and nicknames	**User choice**
Dingwall et al. (74)	Mixed methods	First nations youth	Aboriginal and Islander Mental Health Initiative for Youth (AIMhi-Y)	CBT, psychoeducation, mindfulness	Australia	**Interfaces**: Adding photos of people who care about the user	**User choice**
Edbrooke-Childs et al. (114)	Clustered RCT	General adolescent and youth population	Power Up	Patient activation	UK	**Interfaces:** Customize the iconography as straight-lined or cartoon-style **Communication (non-therapeutic):** Add people to their support network, **Interactivity**: Personalized input methods such as photos, videos, audios and text	**User choice**
Fitzpatrick et al. (78)	RCT	University students	Woebot	Conversational agent, CBT	USA	**Content (therapeutic)** **Communication (non-therapeutic)**: Personalized messages to initiate the conversation **Interactivity**: Goal setting	**User choice** **Rule-based**: Based on mood state
Fleming et al. (94)	Usability testing	Sexual minority men	TODAY!	CBT	USA	**Guidance**: Personalized feedback	**Machine learning**: Based on unique needs of the user
Garrido et al. (79)	Qualitative	University students	MoodyTunes	CBT, experimental learning	Australia	**Content (therapeutic)**: Automatic playlists of sfeel better’ music	**Machine learning**: Based on the user's recorded effect particular pieces of music having on their mood
Gonsalves et al. (63)	Mixed methods	Secondary school students	POD Adventures	Gamification	India	**Content (therapeutic)**: Creating user's own PODs	**User choice**
Gonsalves et al. (64)	Co-design	Secondary school students	POD Adventures	Gamification	India	**Content (therapeutic):** Creating user's own PODs	**User choice**
Grist et al. (53)	Mixed methods	General adolescent and youth population	BlueIce	CBT, dialectical behavioral therapy	UK	**Content (therapeutic)**: Personalized mood-lifting activities **Communication (non-therapeutic)**: Contact list	**User choice**
Harrer et al. (86)^†^	RCT	University students	StudiCare Stress	CBT, Lazarus’ transactional model of stress	Germany	**Guidance**: Personalized feedback on demand **Communication (therapeutic)**: Reminder	**Provider choice**: Feedback by eCoaches
Jones et al. (66)	Mixed methods	General adolescent and youth population	MoodHwb	CBT, positive psychology, behavioral change theory	UK	**Interactivity:** Saving favorites (favorite sites/links to sources such as videos, pictures, music), having user's own goals	**User choice**
Jones et al. (65)	Qualitative	General adolescent and youth population	MoodHwb	CBT, positive psychology, behavioral change theory	UK	**Interactivity:** Saving favorites (favorite sites/ links to sources such as videos, pictures, music), having user's own goals	**User choice**
Kadirvelu et al. (105)	User-centered design	General adolescent and youth population	Mindcraft	Mood tracking	UK	**Content (therapeutic)**: Daily active questions tailored to user's unique mental health objectives **Interactivity**: Select which passive/sensor data to share	**User choice**
Kahl et al. (59)	Longitudinal	General adolescent and youth population	ReachOut.com	Psychoeducation	Australia	**Guidance**: Customized recommendations	**Rule-based**: Based on user responses
Kennard et al. (102)^†^	RCT	General adolescent and youth population	BRITE	Emotion regulation	USA	**Content (therapeutic)**: Distress tolerance strategies, emotion regulation skills and safety plans	**User choice**: User preferences **Provider choice**: Strategies populated by the therapist in collaboration with the participant **Rule-based**: Based on symptom profile and level of distress
Kooiman et al. (97)	Mixed methods	General adolescent and youth population	StayFine	Preventive cognitive therapy, CBT	Netherlands	**Content (therapeutic)**: Content of the modules	**Provider choice**: Shared decision making with therapist **Machine learning**: Based on prior diagnostic classification, Questionnaire responses and EMA
Lattie et al. (80)^†^	Pilot study	University students	IntelliCare	Mood tracking	USA	**Content (non-therapeutic)**: Mental health resources **Guidance**: Personalized feedback	**Rule-based**: Based on user's university location **Machine learning**: Based on symptom elevation
Levin et al. (87)^†^	RCT	University students	Stop, Breathe, and Think	Mindfulness	USA	**Content (therapeutic)**: Personalized therapy exercises	**Machine learning**: Based on user's initial check-in assessments
Litvin et al. (82)	RCT	University students	eQuoo	CBT, systemic psychology, positive psychology, psychoeducation, gamification	UK	**Content (non-therapeutic)**: Choice of the story **Interfaces**: Customized avatar	**User choice**
Liu et al. (89)^†^	RCT	University students	XiaoNan	CBT, conversational agent	China	**Order**: Delivers the therapy content based on user's responses	**Machine learning**: Based on user responses using AI
Lucassen et al. (111)	Quantitative	General adolescent and youth population	SPARX	CBT, serious games	NZ	**Interfaces:** Avatar customization including gender (binary) of the avatar	**User choice**
Malik et al. (71)	Qualitative	Female youth	JoyPop	Resilience theory	USA	**Communication (therapeutic)**: Timer to play sleep sounds **Communication (non-therapeutic)**: Social contacts	**User choice**
McCloud et al. (85)^†^	RCT	University students	Feel Stress Free	CBT	UK	**Content (therapeutic)**: Personalized therapy activities	**Machine learning**: Based on user inputs on “thought trainer” and “mood meter”
McManama O'Brien et al. (101)^†^	Pilot study	General adolescent and youth population	Crisis Care	CBT	USA	**Content (therapeutic)**: Relaxation techniques, choice of activities to engage **Content (non-therapeutic)**: Music, video clips and images **Communication (non-therapeutic)**: Contact list	**User choice**
Mens et al. (51)	Longitudinal	General adolescent and youth population	The Grow It!	CBT, games	Netherlands	**Content (therapeutic)**: Choose one of three unique challenges each day **Interfaces**: Customized user profiles and nicknames	**User choice**
Moor et al. (109)	Feasibility study	General adolescent and youth population	BRAVE-TA	CBT	NZ	**Guidance:** Personalized therapist feedback	**Provider choice**
Neal-Barnett et al. (95)	Mixed methods	Black or biracial seventh- and eighth-grade adolescent females	Build Your Own Theme Song (BYOTS)	Cognitive restructuring, music	USA	**Content (therapeutic):** Personalized theme song	**User choice**
Newman et al. (83)^†^	RCT	University students	*Unnamed* (guided self-help mobile programme)	CBT	USA	**Guidance**: Personalized feedback from coaches	**Provider choice**: Feedback and support from trained coaches
Newton et al. (100)	User-centered design	General adolescent and youth population	MindClimb	Relaxation	Canada	**Content (therapeutic)**: Personalized exposure activities and coping strategies	**User choice**
Nicol et al. (107)	RCT	General adolescent and youth population	W-GenZ (Woebot for Adolescent Depression)	CBT, Interpersonal Psychotherapy for Adolescents (IPT-A), Dialectical Behavior Therapy (DBT)	USA	**Order:** Deliver tailored conversations in real time	**Machine learning:** Based on user's current mood and needs
Ospina-Pinillos et al. (99)	Exploratory	General adolescent and youth population	Mental Health eClinic (MHeC)	Self-assessment	Colombia	**Content (therapeutic)**: Well-being plans, personalized assessment **Guidance**: Mental health app recommendations	**Provider choice**: Recommendations from health professionals **Rule-based**: Rule-based decision algorithms
O’Dea et al. (113)	RCT	General adolescent and youth population	WeClick	CBT	Australia	**Interfaces**: User's own character profile	**Machine learning**: Based on user's activity completion
Peuters et al. (92)	RCT	Secondary school students	#LIFEGOALS	Health Action Process Approach (HAPA) model, Elaboration Likelihood Model (ELM), Persuasive Systems Design model (PSD)	Belgium	**Content (therapeutic)**: Choose own action plans, select when and how to use the intervention **Interfaces**: Customized chat environment/user profile/ avatar with earned rewards	**User choice**
Pozuelo et al. (106)	User-centered design	General adolescent and youth population	Kuamsha	Behavioral activation	sub-Saharan Africa	**Order**: Choose the actions the character takes **Interfaces**: User's character to be a part of the story	**User choice**
Prochaska et al. (108)	Evaluation study	General adolescent and youth population	BeMe	CBT, dialectical behavior therapy, acceptance and commitment therapy, mindfulness-based self-compassion, positive psychology, and motivational interviewing	USA	**Guidance:** Feedback	**Provider choice**
Raevuori et al. (84)^†^	RCT	University students	Meru Health Programme	Mindfulness, CBT, behavioural activation	Finland	**Guidance**: Personalized guidance/assistance from therapists	**Provider choice**: Therapist guidance/assistance
Ribanszki et al. (91)	Qualitative	Secondary school students	Thrive	Guided relaxation, games, mood tracking	UK	**Content (therapeutic)**: Customized cognitive training plan **Interfaces**: Background/avatar customization	**User choice** **Machine learning**: Based on short mood assessment and thought training exercise
Rice et al. (70)^†^	Pilot study	General adolescent and youth population	Rebound	Positive psychology, mindfulness, CBT	Australia	**Guidance**: Personalized suggestions	**Provider choice**: Suggestions from moderators
Santesteban-Echarri et al. (69)^†^	Qualitative	General adolescent and youth population	Rebound	Positive psychology, mindfulness, CBT	Australia	**Guidance**: Personalized suggestions	**Provider choice**: Suggestions from moderators
Shi et al. (58)	RCT	General adolescent and youth population	Thought Spot	Social cognitive theory, theory of help-seeking, mood tracking	Canada	**Content (non-therapeutic)**: Mental health resources **Interactivity**: Save the favourite spot	**User choice** **Machine learning**: Based on geo-location
Silk et al. (98)	Open trial	General adolescent and youth population	SmartCAT 2.0	CBT	USA	**Content (therapeutic):** Customized therapy materials such as exposure assignments **Communication:** Location-aware reminders	**Provider choice:** Customized materials were decided by the therapists based on user's progress **Rule-based:** Geo-fencing for location-aware reminders
Six et al. (88)^†^	RCT	University students	AirHeart App	CBT	USA	**Interfaces**: Customized avatar and app elements, personalized journal and mood tracking chart to user's name	**User choice**
Stallard et al. (54)	Open trial	General adolescent and youth population	BlueIce	CBT, dialectical behavioral therapy	UK	**Content (therapeutic)**: Personalized mood-lifting activities **Communication (non-therapeutic)**: Contact list	**User choice**
Stallard et al. (55)	RCT	General adolescent and youth population	BlueIce	CBT, dialectical behavioral therapy	UK	**Content (therapeutic):** Personalized mood-lifting activities **Communication (non-therapeutic):** Contact list	**User choice**
Tighe et al. (62)	RCT	Indigenous youth	ibobbly	Acceptance-based therapy, self-assessment	Australia	**Content (therapeutic)**: Personalized action plan **Content (non-therapeutic)**: Personalized dashboard indicating user's progress	**Machine learning**: Based on self-assessment data and user's goals
Tighe et al. (61)	Pilot study	Indigenous youth	ibobbly	Acceptance-based therapy, self-assessment	Australia	**Content (therapeutic)**: Personalized action plan **Content (non-therapeutic)**: Personalized dashboard indicating user's progress	**Machine learning**: Based on self-assessment data and user's goals
van Doorn et al. (81)	Mixed methods	University students	ENYOY-Sense IT	Bio cueing	Netherlands	**Content (therapeutic)**: Personalized smartwatch **Communication (therapeutic)**: Personalized frequency and content of the notifications	**User choice** **Rule-based**: Based on symptom profile
Viduani et al. (110)	Mixed methods	General adolescent and youth population	IDEABot	Conversational agent	Brazil	**Communication (therapeutic)**: Reminders	**User choice**
Werner-Seidler et al. (68)	RCT	General adolescent and youth population	Sleep Ninja	CBT	Australia	**Interactivity**: Save and revisit favorite sleep tips	**User choice**
Werner-Seidler et al. (67)	Pre-post study	General adolescent and youth population	Sleep Ninja	CBT	Australia	**Interactivity:** Save and revisit favorite sleep tips	**User choice**
Wiljer et al. (56)	RCT	University students	Thought Spot	Social cognitive theory, theory of help-seeking, mood tracking	Canada	**Content (non-therapeutic)**: Mental health resources **Interactivity**: Save the favorite spot	**User choice** **Machine learning**: Based on geo-location
Wong et al. (57)	Qualitative	University students	Thought Spot	Social cognitive theory, theory of help-seeking, mood tracking	Canada	**Content (non-therapeutic)**: Mental health resources **Interactivity**: Save the favorite spot	**User choice** **Machine learning**: Based on geo-location
Wozney et al. (96)^†^	Mixed methods	General adolescent and youth population	Breathe	CBT	Canada	**Content (therapeutic)** **Communication (therapeutic)**: Personalized reminders	**Rule-based**: Based on user responses or demographic information

^†^ Papers retrieved from review articles.

### 3.1 Study characteristics

**Figure 4:**
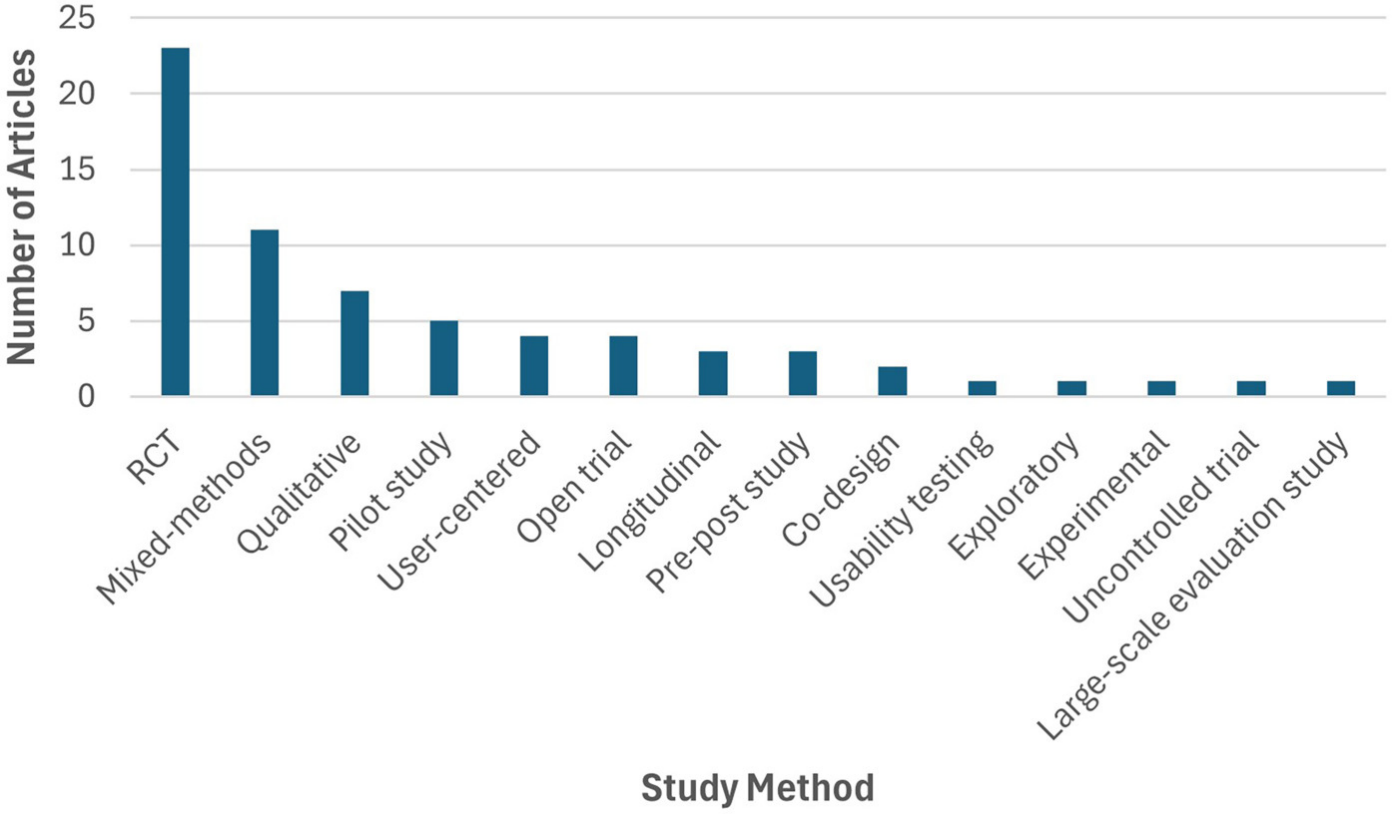
Distribution of research methods used among the selected articles.

The chosen studies have employed diverse methodologies for user data collection, with randomized controlled trials (RCTs) emerging as the prevalent choice, closely trailed by mixed-method studies which employed both qualitative and quantitative research designs. Qualitative study designs were also employed to investigate user experiences with various approaches such as semi-structured or focus-group interviews or user surveys. At lesser frequency, pilot studies were conducted to assess the feasibility and acceptability of the interventions, followed by user-centered study designs to incorporate user feedback and open trials. Apart from these prevailing research designs, longitudinal studies, pre-post studies, co-designs, usability testing, exploratory, experimental, uncontrolled trials and large-scale evaluation studies (of acceptability and utility) were also observed. Figure 4 illustrates the distribution of methodologies adopted across the selected articles.

Among the studies concentrating on a single application or intervention, Cognitive Behavioral Therapy (CBT) emerged as the most frequently utilized therapeutic technique. Additionally, mood tracking, positive psychology, psychoeducation, acceptance-based therapy, self-assessment, mindfulness, games or gamification, and behavioral change and activation were each employed in more than two instances within the selected articles.

Some apps were used more than once in the selected interventions. Both the apps “The Grow It!” (48–51) and “BlueIce” (52–55) were used in four interventions. “Thought Spot” (56–58) was used in three interventions. “Reachout.com” (59, 60), “ibobbly” (61, 62), POD Adventures (63, 64), MoodHwb (65, 66), Sleep Ninja (67, 68), Rebound (69, 70) and JoyPop (71, 72) were each utilized in two studies.

**Figure 5:**
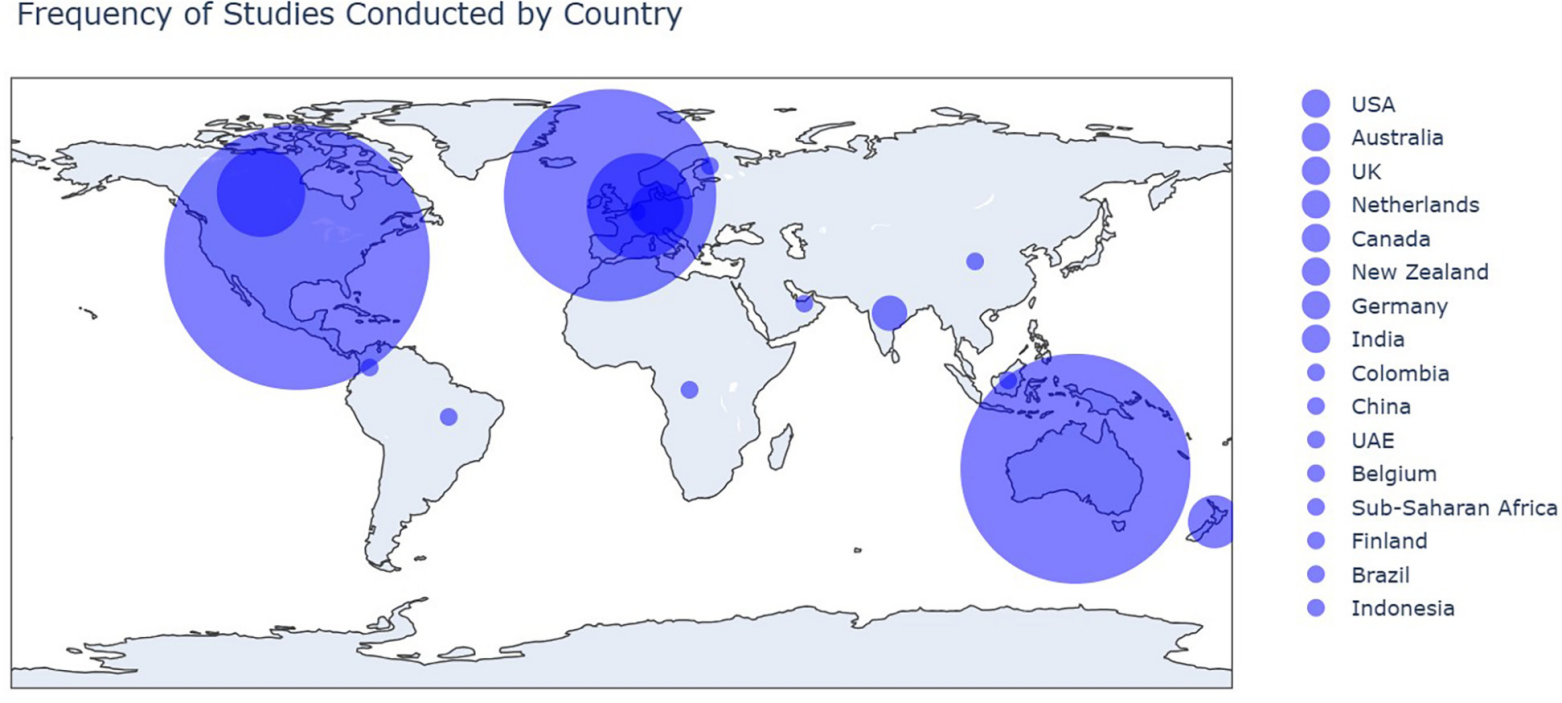
Geographical distribution of studies across countries.

The largest number of studies was conducted in the United States of America (USA), followed closely by Australia, and the United Kingdom (UK), and then the Netherlands and Canada. Conversely, only six studies were conducted outside of North America, Oceania, and Europe regions. Figure 5 depicts the distribution of studies categorized by country.

When selecting participants for the studies, only five studies specifically targeted indigenous or First Nations individuals (61, 62, 72–74). Eighteen studies focused on recruiting university students (52, 56, 57, 75–89), and six studies aimed to recruit secondary school students (63, 64, 90–93). One study was conducted with sexual minority men (94), while another study exclusively recruited females (71). One study recruited “black or biracial seventh- and eighth-grade adolescent females” (95). The remaining studies targeted general adolescent or youth populations.

### 3.2 Personalization

This section examines the personalization aspects in the selected articles, focusing on dimensions and mechanisms as defined above. It discusses the types of dimensions and mechanisms reported, as well as the frequency of their application in the interventions.

#### 3.2.1 Personalization dimensions

Among the retrieved studies, about half of the interventions (34 of 67, ∼51%) used only one personalization dimension. However, 29 of the 67 interventions (∼43%) used two dimensions, and 3 interventions used three dimensions (4%). Personalization of therapeutic content was the most commonly applied dimension, used by 34 interventions (∼51%). Various forms of personalization of therapeutic content were applied, including customization of module content (48, 51, 78, 96–98), personalized action plans and cognitive training plans (50, 61, 62, 91, 92, 99), personalized therapeutic exercises, activities, and coping strategies (52–55, 75–77, 85, 87, 93, 100–103), and personalized assessments (90, 99, 104). Further, Garrido et al. (79) offered the users personalized “feel better” music, while Neal-Barnett et al. (95) allowed users to create their own theme song to cope with negative thoughts. POD Adventures app (63, 64) allowed the users to have their own PODs (Problem identification, Option generation and Do it plan) to solve their own problems. Kadirvelu et al. (105) tailored personalized therapeutic questions each day during the intervention, and van Doorn et al. (81) offered a smartwatch to their participants which could be personalized.

Personalization of non-therapeutic content was also employed by 8 interventions (12%). Personalized information relating to available mental health resources based on the user's geo-location was applied by the Thought Spot app (56–58) and IntelliCare app (80). The ibobbly app (61, 62) provided users with a personalized dashboard indicating their progress. Users were also given the ability to have their own music, video clips, and images (101) and choose the game story of the intervention to proceed with the therapy (82).

Order is the least used personalization dimension, applied by only 3 interventions (4%) (89, 106, 107). The intervention by Pozuelo et al. (106) allowed users to have their own character and interactively choose the actions the character undertakes within the story. The AI chatbot XiaoNan developed by Liu et al. (89) illustrates personalized CBT responses based on the user's intent and emotions. The W-GenZ (Woebot for Adolescent Depression) app (107) deliver tailored conversation in real-time based on the user's current mood and needs.

Personalized guidance provided by the app/website of the intervention or by coaches, therapists, and moderators in blended interventions was evident in 14 interventions (21%). Mainly this took the form of personalized recommendations, suggestions, and assistance on therapy exercises and activities (59, 69, 70, 75, 84). Personalized feedback was also given to users (80, 83, 86, 94, 108, 109), and mental health apps were recommended (60, 99). Further, the ExpDepression app automatically referred its users to a care team, suicide help-line, crisis manager or entrusted caregiver based on risk categories and self-harm behavior (104).

Personalized communication on therapeutic elements (10%) of the intervention is provided by personalizing the frequency and content of therapy notifications (81), personalized reminders (86, 96, 98, 110), and allowing users to set a timer to play sleep sounds (71, 72). Non-therapeutic communication (12%) was personalized by allowing users to have their own contact list/social contacts (52–55, 71, 72, 101) and by delivering personalized messages to initiate conversations with the user (78).

DMHI interfaces can support customization of non-therapeutic elements such as app background and avatar. Seventeen interventions (25%) used this dimension to personalize user experience. These interventions were observed to incorporate avatar, game characters, app background, chat environments, and color scheme customization (73, 82, 88, 91–93, 106, 111, 112) and facilitating personalized user profiles with nicknames, character profiles, and mood profiles (48–51, 88, 92, 113). Lucassen et al. (111) specifically facilitated the users to choose a preferred gender (binary) of their avatar while allowing them to customize it. Customization of gamified elements and content, such as personalized progress levels, rewards, and the ability to customize the “home island” in a user's quest, was also evident (73, 93). Edbrooke-Childs et al. (114) introduced customizable iconography so the users can choose between straight-lined or cartoon-styled icons. One intervention allowed users to add photos of people who care about them the most (74).

Personalized interactivity involves customizing non-therapeutic elements that users want to revisit, reuse, or use to interact with the intervention in different ways. Overall, eleven studies (16%) investigated personalized interactivity. Allowing users to save their favorite elements, such as locations of mental health services, websites, sleep tips, app contents and links to resources like videos, pictures, and music, was considered by some interventions (56–58, 65–68, 76). Three interventions facilitated users in setting their own goals (65, 66, 78), and one intervention allowed users to select the passive/sensor data they wished to share (105). The Power Up app (114) allowed users to add people to their support network to share app content. It also facilitated users to interact with the app with different input methods such as photos, videos, audio or text.

#### 3.2.2 Personalization mechanisms

From the 67 studies gathered for this review, 53 (∼79%) employed only one personalization mechanism. However, 12 studies (∼18%) utilized two mechanisms, with 5 of these combining user choice with machine learning techniques. Three studies combined provider choice with rule-based methods while 2 studies explored the combination of user choice with rule-based mechanisms. Two studies investigated combining machine learning mechanisms with rule-based and provider choice. Additionally, two studies combined user choice, provider choice, and rule-based methods to enhance personalized experiences.

User choice is the most commonly used personalization mechanism applied by 40 interventions out of 67 (∼60%). Provider choice was used by 13 interventions (∼19%), where recommendations/guidance/assistance were delivered by health professionals (83, 84, 86, 97, 99, 102, 108, 109), sometimes facilitating shared-decision-making with the therapists (97), or suggestions were offered by the moderators (69, 70). Further, Silk et al. (98) and Atik et al. (76) investigated tailoring intervention content and tasks to users based on their progress, with therapists making the adjustments. Meanwhile Chen et al. (104) delivered a modified PHQ-9, informed by professionals’ assessments, to ExpDepression users.

Ten interventions used rule-based mechanisms (∼15%) to personalize the user experience. Rule-based automation was focused based on user responses (59, 96), symptom profile (81, 102), mood state (78) or user's university location (80). The Elona Therapy app (76) took symptom profiles, user needs, diagnoses, therapy progress, and joint therapy decisions into account when using rule-based automation to deliver personalized therapy materials. Silk et al. (98) used geo-fencing to identify users' locations and send location-aware reminders. When a user enters a location where they tend to experience anxiety, the app sends a reminder to use it. Chen et al. (104) categorized users' risk levels based on their mood, PHQ-9 scores, and self-harm ideation, while, Ospina-Pinillos et al. (99) reportedly used “rule-based decision algorithms” to enable personalized assessment of the users.

Machine learning mechanisms were the second most common method for delivering personalization among the gathered studies. Twenty out of 67 interventions (∼30%) used various types of machine learning mechanisms. The Thought Spot app uses the user's geo-location to provide information about mental health services that are located nearer to the user (56–58). Some interventions have used user's self or real-time mood assessments to personalize the experience (49, 61, 62, 87, 103, 107). User responses on app contents or questionnaires (79, 86, 89, 90) were also considered when personalizing the user experience. Specifically, Garrido et al. (79) captured the recorded effect some music pieces have on the user's mood to automatically generate a “feel good” music playlist. And Burn et al. (90) have used computerized adaptive testing (CAT) driven by an algorithm to select real-time questions based on user's ongoing responses. A few studies have taken users' goals and unique needs to generate personalized action plans or provide personalized feedback (61, 62, 94), while personalization based on activity completion (113), symptom elevation (80) and heart rate (75) were also evident. Kooiman et al. (97) have used a multimodal data-driven personalization approach, which considered prior diagnostic classification, self-report questionnaire responses and ecological momentary assessment (EMA) to personalize the content of the modules. Bidargaddi et al. (60) incorporated the user's selection of goals and answers to interactive quizzes to generate personalized app recommendations, while Ribanszki et al. (91) considered a short mood assessment and thought training exercises to deliver personalized cognitive training plans to the user.

## 4 Discussion

This review answers the research question (“which personalization variables are utilized in DMHIs designed for adolescents and youth?”) that was posed in the introduction. In order to give a comprehensive consideration of personalization elements, this review extended the conceptual framework proposed by Hornstein et al. (44) and explored how the defined dimensions and mechanisms were employed in recent DMHIs for anxiety and depression in adolescents and youth. Apart from the main findings, the review further investigated the characteristics of studies that concentrated on personalization, as well as the demographics of the participants.

### 4.1 Study characteristics

The findings of the study characteristics showed where the DMHIs included in this review were conducted, the methods they used, the techniques they employed, and the characteristics of the participants. The selected studies for this review were mainly conducted in America, Australasia, and Europe. In recent years, studies that used personalization in DMHIs for depression and anxiety in adolescents and youth have been limited to only four studies on the Asian continent. When compared to America, Australasia and Europe, Asia exhibits lower or decreasing age-standardized incidence rates of depression in adolescents/youth aged 10–24 years (115). This trend may have influenced America, Australasia and Europe regions to increase their research on adolescent mental health compared to Asia. However, in light of total population, Asia and Africa need to be considered as under-studied in this domain.

With respect to the characteristics of the participants, only one study considered sexual minority men. Otherwise, rainbow users were unaddressed even though it is reported that transgender and gender-diverse youth experience considerable mental health disparities when compared with their cisgender peers, including higher rates of depression, anxiety, and suicidality (116). Although this review does not cover all DMHIs for depression and anxiety in adolescents and youth, the lack of research on personalized user experiences on DMHI engagement for gender-diverse young populations is notable. Comparatively, the presence of studies with indigenous and First-Nations young people in the findings shows at least some attention to DMHIs for these communities. Furthermore, only one study specifically investigated the impact of DMHIs on Black or biracial females (95). Black and multiracial adolescents face the highest risk of suicide among US college students (117) but are the least likely to access preventive therapy (118). Research suggests that mental health providers may be reluctant to acknowledge their role in perpetuating racial disparities in care (119). Therefore, the findings of this review provide further evidence of this reluctance, highlighting the need for future research to improve DMHIs tailored to multiracial adolescents and youth.

Almost all the apps and websites that incorporate personalization among the reviewed interventions were non-commercialized. There were no findings addressing the effect of personalization of commercialized mental health apps such as Headspace, Calm or Replika on youth depression and anxiety. The barrier to the usage of commercialized apps in scientific interventions could be their high subscription fees and freemium models or licensing constraints. Also, having strategies like freemium negatively affects user experiences specifically for vulnerable users (120). Therefore, interventions tend to use either a free version of commercialized apps (121) or shift their focus to non-commercialized apps.

### 4.2 Personalization

Personalization has been suggested to be effective in increasing adherence to DMHIs among youth (122, 123). This review investigates what has been personalized and how the personalization has been applied in recent DMHIs for adolescents and young people with depression and anxiety. The review adapted the conceptual framework of Hornstein et al. (44) and modified it to categorize both the therapeutic and non-therapeutic personalization elements discovered in the selected interventions. Considering non-therapeutic personalization elements, Hornstein et al. (44) “content” and “communication” dimensions have been extended and two new personalization dimensions, namely “interfaces” and “interactivity”, have been added. Conversely, the definitions of personalization mechanisms have been taken as defined by the original framework.

Personalizing therapeutic content was largely evident in this review. Yet, facilitating change in the order of the sessions or modules was weakly addressed. Among the sixty-seven articles, only three (89, 106, 107) have reported enabling personalization of the order of therapeutic sessions of modules (4%). A similar observation was made by Hornstein et al. (44), who also found that 4% of the interventions enabled personalization of the order. Nevertheless, even though this aspect of personalization is underexplored, tailored sequencing of modules specific to the type of mental health disorder, especially when interference such as comorbid conditions and stressors occur, has been found to be effective with youth (124). Further, some study participants have argued for the convenience of tailored sequencing of modules to one's needs as the interventions could become less attractive if they already understood the concepts but still have to follow the order of modules to complete (125). Therefore, more thorough investigation is needed to understand the possibility of applying personalization of the module sequences in DMHIs for youth and why it has not been widely applied.

The review found that personalization of DMHI interfaces was mainly focused on customizing the appearance of avatars such as clothing or hairstyles. However, offering users the ability to personalize other characteristics of an avatar such as gender or incorporating gender-diverse characters in game-based interventions could enhance user engagement, as discussed in recent studies (45, 126). However, only one study provided users with the ability to customize their avatar's gender (111), though the options were limited to a binary selection. Yet, employing gender as a characteristic to personalize the experience was also not visible in the selected studies for this review. The reluctance to employ gender in therapeutic or non-therapeutic personalization dimensions could be caused by the recent recognition of gender as a spectrum instead of binary and the recognition of the importance of inclusivity and diversity in research and design (127, 128). Using gender as a variable to personalize user experience may inadvertently exclude or marginalize individuals who do not identify with the provided options.

The lack of generative AI in the retrieved studies is notable. Machine learning was widely used to personalize therapeutic and non-therapeutic elements in the studies. Yet, the study incorporating the greatest range of AI personalization (89) was still a largely template-and-rule based method. While the rapidly growing field of generative AI holds significant potential for widespread use in digital mental health interventions—ranging from mental disorder detection and counselling support to therapeutic applications, clinical training, decision-making support, and goal-driven optimization—its inherent limitations underscore its role as a supplementary tool rather than a replacement for mental health professionals (129). Further, generative AI could be used in DMHIs to provide personalized experiences to users with conversational support, mood predictions, and risk assessments (130), and it could assist mental health practitioners in re-defining mental illnesses more objectively and personalizing the treatment based on user characteristics (131). Despite the potential benefits of AI in this domain, lack of sufficient data to train the models (132), ethical challenges such as biased data (e.g., instinctive and expressive quality of clinical text data, connecting mental disorders to specific ethnic groups, etc.) (133) and lack of established standards to guide the use of AI in healthcare settings (134) could be among the reasons for hesitation in AI use with DMHIs. Ameliorating these challenges with standard practices to produce clinical data (135) and use AI in healthcare (136) would be beneficial for an enhanced user experience.

Automated mechanisms such as rule-based and machine learning in this review mainly take into account user's symptom profile, mood state and responses to pre-defined questionnaires. There is a lack of evidence on using user characteristics such as culture, age, ethnicity and personality. However, these characteristics were evidently employed in DMHIs in general (40, 137, 138). Although culture has not been directly recognized as a personalization variable, Thabrew et al. (139) observed positive feedback on using features to increase cultural appeal such as introductory “karanga” (Māori ceremonial welcome call). Hence, culturally appropriate content could be adapted to enhance user engagement. Further, this review being narrowed in its scope to adolescents and youth could be pointed out as a reason why age is rarely recognized as a variable that was used to personalize the user experience. However, one study in this review (114) used customizable iconography, allowing users to choose between a straight-lined or cartoon-styled design. Within the 11–19 age range, the straight-lined style was aimed at older adolescents, while the cartoon-styled design targeted younger adolescents. Such segmentation was also observed in the study by Fleming et al. (138), which found that perceptions of gaming contexts differ between younger and older adolescents as younger adolescents tend to prefer interactive and gamified interventions while older adolescents preferred straightforward and concise designs. Therefore, DMHIs for adolescents and youth could still use age as a variable to personalize their engagement. Personalizing user experience based on user's ethnicity may not have been evident in this review because, similar to gender, ethnicity or race is a complex social construct with significant variation within and across racial groups. Therefore, treating ethnicity as a monolithic variable oversimplifies individuals' experiences and identities, ignoring the diversity and intersectionality of race with other aspects of identity. Further, a recent study (40) witnessed that understanding the user's evolving personality traits can help gauge their receptiveness to targeted content, taking into account factors like competitiveness or openness.

## 5 Future research directions and study limitations

Among the selected interventions in this study, personalization was not a core focus. However, given the well-documented challenges of engagement and adherence in DMHIs for adolescents and young people, there is a strong rationale for further exploring personalization as a means to address these issues. Greater emphasis on evaluating the effectiveness of personalization elements, particularly in terms of their influence on user engagement and long-term adherence, could provide valuable insights into optimizing DMHIs for this population.

As highlighted in the Discussion section, there is room for improvement in personalization within DMHIs, particularly for this demographic. One promising area for future research is the use of generative AI and large language models (LLMs) to enhance the personalization of user experiences. Further studies could explore the establishment of standard practices for employing AI in healthcare settings. Additionally, research could expand on how engaging user characteristics such as age, gender, and ethnicity can provide a more personalized experience. The possibility of personalizing the sequence of intervention modules for youth also warrants further investigation.

Although this review reveals a broader spectrum of personalization, some elements that were evidently used in recent DMHIs not specifically for adolescents could not be observed in this review. A few of them could be listed as integration of video games into specific clinical care processes (126), personalized session scheduling (140), inclusion of personalized information regarding the app's purpose and terms of use as an additional safety measure (141), personalized trigger warnings (142) and predictive modelling to identify individuals at risk and proactively provide them with targeted interventions (143). Therefore, future DMHIs for adolescents and youth with depression and anxiety could consider these unaddressed areas for enhanced adherence to mental health interventions and user engagement.

This review has several limitations that should be acknowledged. It was not pre-registered, which is a common practice to enhance transparency and reduce potential bias. However, we followed a structured and systematic approach aligned with established scoping review methodologies to ensure rigor and comprehensiveness. Also, this review was based solely on peer-reviewed articles in English, specifically targeting DMHIs for adolescents and youth with anxiety and depression. Consequently, interventions from commercial settings, non-English sources, or those addressing other mental health issues may be underrepresented or entirely excluded. The restriction to English may partially account for the lack of retrieved studies from Asia. Additionally, the limited descriptions of intervention features in many of the included articles made it challenging to thoroughly assess personalization elements. To mitigate this, we tracked down and reviewed available study protocols and related publications. Despite these efforts, some personalization aspects of the interventions may still be missing from this review.

## 6 Conclusion

This review was conducted to understand what has been personalized in DMHIs for adolescents and young people and how this personalization is applied. It adapts the conceptual framework proposed by Hornstein et al. (44) and expands it with non-therapeutic personalization elements. The results of the review indicate that therapeutic content is the most common object of personalization, and that interventions favor user choice as the personalization mechanism. Although incorporating personalization elements in DMHIs has the potential to improve them, there is insufficient investigation into its impact on user experience and adherence among adolescents and young people. Additionally, while machine learning techniques have been frequently employed to personalize user experience, the limited use of generative AI and LLMs is noteworthy. Future studies could address these research gaps to enhance the experience of young people in DMHIs, ultimately contributing to more effective mental health interventions and policies.

## Data Availability

The original contributions presented in the study are included in the article/Supplementary Material, further inquiries can be directed to the corresponding author.
